# Expression of CCL2, FOS, and JUN May Help to Distinguish Patients With IgA Nephropathy From Healthy Controls

**DOI:** 10.3389/fphys.2022.840890

**Published:** 2022-04-07

**Authors:** Xue Zhou, Ning Wang, Yuefeng Zhang, Pei Yu

**Affiliations:** ^1^NHC Key Laboratory of Hormones and Development, Chu Hsien-I Memorial Hospital and Tianjin Institute of Endocrinology, Tianjin Medical University, Tianjin, China; ^2^Tianjin Key Laboratory of Metabolic Diseases, Tianjin Medical University, Tianjin, China; ^3^Department of Nephrology, Tianjin Haihe Hospital, Tianjin, China; ^4^Tianjin Third Central Hospital, Tianjin, China

**Keywords:** IgA nephropathy, diagnosis, biomarker, immune, bioinformatics analysis

## Abstract

**Background:**

IgA nephropathy (IgAN), the most common type of glomerulonephritis worldwide, can only be diagnosed mainly by renal biopsy owing to lack of effective biomarkers. It is urgent to explore and identify the potential diagnostic biomarkers through assessing the gene expression profiles of patients with IgAN.

**Methods:**

Two datasets were obtained from the Gene Expression Omnibus (GEO) database, including GSE115857 (55 IgAN, 7 living healthy donors) and GSE35487 (25 IgAN, 6 living healthy donors), then underwent differentially expressed genes (DEGs) and function enrichment analyses utilizing R packages. The common gene list was screened out between DEGs and immune-associated genes by Venn diagram, then performed gene-gene interaction, protein-protein interaction (PPI) and function enrichment analyses. Top three immune-associated hub genes were selected by Maximal Clique Centrality (MCC) method, then the expression and diagnostic value of these hub genes were determined. Consensus clustering algorithm was applied to conduct the unsupervised cluster analysis of the immune-associated hub gene list in IgAN. Finally, the Nephroseq V5 tool was applied to identify the expression level of CCL2, FOS, JUN in kidney diseases, as well as the correlation between CCL2, FOS, JUN expression and renal function in the patients with IgAN.

**Results:**

A total of 129 DEGs were obtained through comparing IgAN with healthy controls *via* the GSE115857 and GSE35487 datasets. Then, we screened out 24 immune-associated IgAN DEGs. CCL2, JUN, and FOS were identified as the top three hub genes, and they were all remarkably downregulated in IgAN. More importantly, CCL2, JUN, and FOS had a high accuracy [area under the curve (AUC) reached almost 1] in predicting IgAN, which could easily distinguish between IgAN patients and healthy individuals. Three distinct subgroups of IgAN were determined based on 24 immune-associated DEGs, with significant differences in the expression of CCL2, JUN, and FOS genes. Finally, CCL2, FOS, JUN were manifested a meaningful association with proteinuria, glomerular filtration rate (GFR), and serum creatinine level.

**Conclusion:**

In summary, our study comprehensively uncovers that CCL2, JUN, and FOS may function as promising biomarkers for diagnosis of IgAN.

## Introduction

IgA nephropathy (IgAN), the most common type of primary glomerulonephritis worldwide (Schena and Nistor, 2018), is caused by the deposition of immune complex at the glomerular mesangial region leading to inflammation and renal failure (Jiang et al., 2016; Su et al., 2017). Approximately 15∼40% of IgAN patients experience deterioration in renal function after 10–20 years of diagnosis and ultimately result in end-stage renal disease (ESRD) (Amico, 2000; Lee et al., 2014). Primary IgAN is an autoimmune disorder with poorly understood etiology, of which immune pathogenesis has been widely studied and reported (Coppo, 2017; Suzuki, 2019). Recent study evidence has confirmed that the histopathological feature of IgAN is the deposition of immune complexes formed by galactose-deficient IgA1 (Gd-IgA1) and corresponding antibody in the mesangial region of the kidney leading to secondary kidney damage, hematuria, proteinuria and other clinical manifestations (Leung et al., 1999). Meanwhile, the deposition of polymeric IgA1 in kidney mesangium in IgA nephropathy patients can increase the synthesis of several inflammatory cytokines such as TNF-α and IL-6 (Leung et al., 2008). The susceptibility and clinical manifestation of IgAN can be affected by genetic polymorphisms concerned immunity and inflammation (Gorgi et al., 2010; Zhang D. et al., 2017; Yang et al., 2018). Notably, immune characteristics, including immunoreactivity and immune cells infiltration in renal tissues, may contribute to uncovering pathological mechanism in the development and progression of IgAN.

At present, IgAN can only be diagnosed by renal biopsy, and there are no effective serum and urine diagnostic biomarkers (Kidney Disease: Improving Global Outcomes [KDIGO] Glomerular Diseases Work Group, 2021). Moreover, owing to the lack of early diagnosis and effective treatment, many IgA nephropathy patients perform poor clinical outcomes. More importantly, understanding the underlying molecular mechanisms contributes to providing guidance to the development of diagnostics approaches. This has raised our interest in seeking the novel potential biomarkers for the early and accurate diagnosis of IgAN patients.

In the current study, we first explored the differentially expressed genes (DEGs) between IgAN patients and healthy controls based on the datasets GSE115857 and GSE35487. A total of 129 DEGs were recognized, of which 24 DEGs were associated with immune. We further determined the top three hub genes as the potential biomarkers such as CCL2, JUN, and FOS, and compared the expression level of these genes in IgAN. In addition, we identified the diagnostic value of the three biomarkers in distinguishing between IgAN and healthy individuals. Due to individual differences in the cases of IgAN, subgroup analysis was performed according to the unsupervised cluster analysis. This is a comprehensive analysis for seeking the potential biomarkers for diagnosis of IgA nephropathy.

## Materials and Methods

### Data Acquisition

The gene expression data of IgAN patients and healthy controls was collected from the Gene Expression Omnibus (GEO) database.^1^ Two datasets (GSE115857 and GSE35487) were collected in our study. The platform for GSE115857 was GPL14951 (Illumina HumanHT-12 WG-DASL V4.0 R2 expression beadchip), which contained 55 IgAN samples and 7 healthy living donors. The platform for GSE35487 was GPL96 (Affymetrix Human Genome U133A Array), which contained 25 IgAN samples and 6 healthy living donors. After normalized conversion of all probes based on the platform annotation information, gene symbols were obtained. Probes corresponding to more than one gene were removed and the average value could be applied for the case of genes corresponding to more than one probe. The “limma” (Ritchie et al., 2015) and “sva” (Parker et al., 2014) packages were utilized to remove batch effect.

### Differentially Expressed Genes Analysis

Differentially expressed genes between IgAN patients and healthy controls were determined with setting a threshold of adjusted *P* < 0.05 and Fold Change > 2 utilizing “Limma” package (Ritchie et al., 2015). Then, these DEGs were visualized by volcano plots and heatmaps.

### Functional Enrichment Analysis

Gene Ontology (GO) and Kyoto Encyclopedia of Genes and Genomes (KEGG) pathway enrichment analyses were achieved *via* “ggplot2,” “GO plot,” and “Cluster Profiler” packages.

### Screening Immune Gene and Identification of Immune-Associated Differentially Expressed Genes

A total of 1793 immune-associated genes were obtained from Immport Shared Data platform.^2^ Venn diagram was applied to perform the intersection between IgAN DEGs and immune-associated gene list, then immune-associated DEGs were identified.

### Construction of Gene-Gene and Protein–Protein Interaction Network of Immune-Associated Differentially Expressed Genes

Genemania^3^ is a web portal for creating gene-gene interaction network. Protein--protein interaction (PPI) network can be created *via* the STRING website^4^ with setting the main parameter of minimum required interaction score [“Low confidence (0.150)”]. Then, the visualization of PPI network was achieved utilizing Cytoscape (version 3.8.0).

### Identification of Diagnostic Biomarkers

Top three immune-associated hub genes, diagnostic biomarkers for IgAN, were obtained by Maximal Clique Centrality (MCC) method *via* Cytoscape. We compared the expression level of the three hub genes between IgAN and healthy controls.

### Diagnostic Value Analysis

The receiver operating characteristic (ROC) curve was applied to evaluate the diagnostic value of the three hub genes in IgAN, which was plotted *via* the “pROC” package. Then, we calculated the value of area under the curve (AUC) (0.5–1). The higher the AUC value is, the better the diagnostic value is. Generally, AUC value in 0.5–0.7 indicates a low effect, AUC value in 0.7–0.9 indicates a middle effect, and AUC value above 0.9 indicates a high effect.

### Unsupervised Cluster Analysis of Immune-Associated Differentially Expressed Genes in IgAN

We normalized the expression data from GSE35487 and GSE115857, and merged them together. Then, the data of 80 IgAN samples was obtained. Based on immune-associated DEGs, different IgAN subgroups were identified utilizing unsupervised cluster analysis *via* “ConsensusClusterPlus” R package. The clusters were generated by the consensus clustering algorithm with running 1,000 times (Wilkerson and Hayes, 2010). Principal component analysis (PCA) was conducted *via* “PCA” R package. We explored the expression level of the three hub genes in the different clusters.

### Clinical Correlation Analysis

The Nephroseq V5 tool was applied to identify the expression level of CCL2, FOS, JUN in kidney diseases, including IgAN, lupus nephritis, diabetic nephropathy, focal segmental glomerulosclerosis, minimal change disease, and membranous glomerulonephropathy. Meanwhile, the correlation between CCL2, FOS, JUN expression and renal function was investigated in the patients with IgAN. Subsequently, the visualization was achieved utilizing the “ggplot2” package.

### Statistics Analysis

Wilcoxon rank sum test was applied to compare the different expression of hub genes. All R packages mentioned above were operated under R software version v4.0.3, and statistical significance was acknowledged in case of *P* < 0.05.

## Results

### Schematic Diagram of the Study Design

**FIGURE 1:**
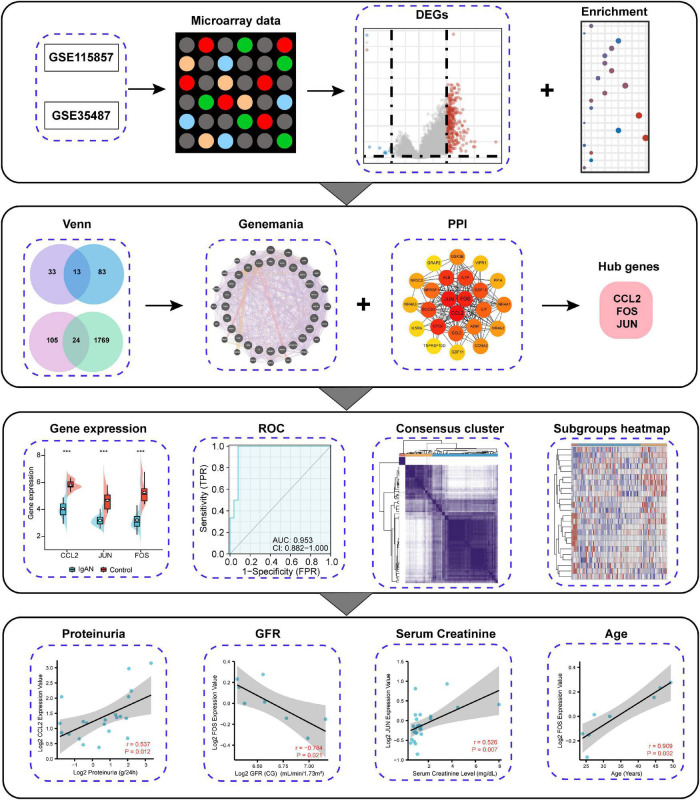
Schematic diagram of the study design.

Since immune complex deposition exerts a role in the occurrence of IgAN, we aimed to comprehensively analyze and identify immune-associated biomarker for the diagnosis of IgAN. The research strategy is presented in Figure 1. We first explored DEGs between IgAN and healthy individuals by means of microarray data. Then, we screened out immune-associated IgAN DEGs and created visual gene-gene and PPI network. The top three hub genes were eventually obtained, which could server as potential biomarkers for IgAN owing to high accuracy of diagnostic value. Furthermore, we conducted IgAN subgroup analysis for an in-depth study. Finally, the correlation between CCL2, FOS, JUN expression and renal function was analyzed in IgAN.

### Identification of Differentially Expressed Genes and Biological Function

**FIGURE 2:**
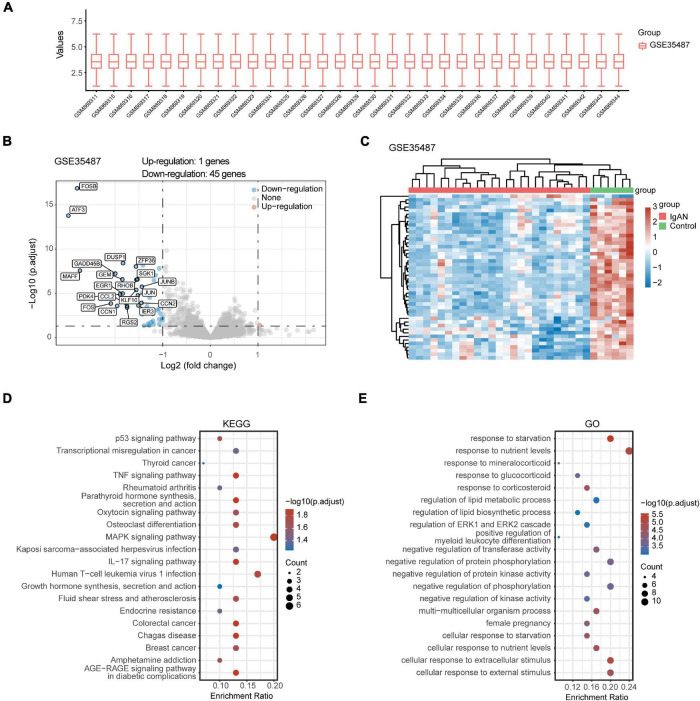
Comprehensive analysis of DEGs and function enrichment in GSE35487. **(A)** Box plot after data standardization. **(B)** Volcano plot of DEGs between IgAN patients and healthy controls. **(C)** Expression heatmap of screened DEGs. **(D)** KEGG and **(E)** GO enrichment analysis of DEGs.

**FIGURE 3:**
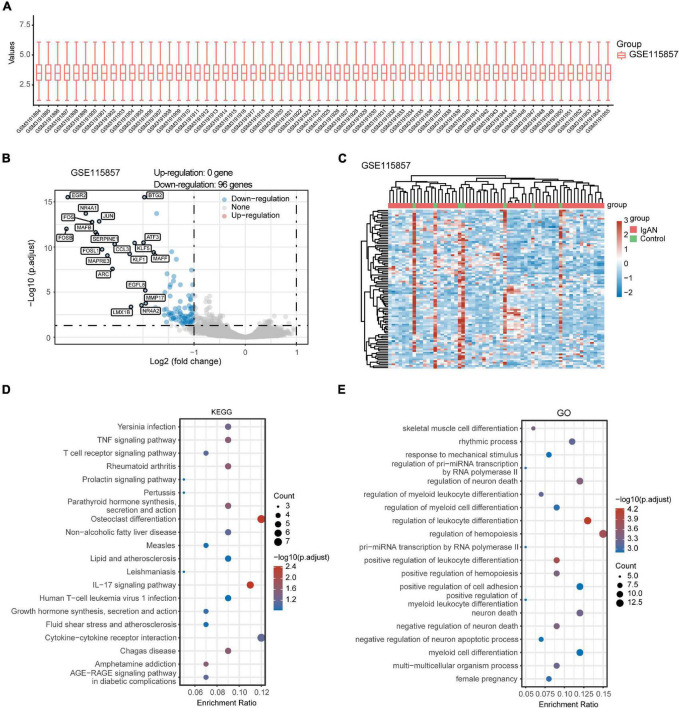
Comprehensive analysis of DEGs and function enrichment in GSE115857. **(A)** Box plot after data standardization. **(B)** Volcano plot of DEGs between IgAN patients and healthy controls. **(C)** Expression heatmap of screened DEGs. **(D)** KEGG and **(E)** GO enrichment analysis of DEGs.

After standardizing the microarray data of GSE35487 (Figure 2A) and GSE115857 (Figure 3A), we identified a total of 46 DEGs in GSE35487 (Figure 2B) and 96 DEGs in GSE115857 (Figure 3B). Subsequently, hierarchical clustering analysis was conducted for displaying DEGs by heatmap respectively (Figures 2C, 3C). Additionally, KEGG and GO enrichment analyses were performed for further research. KEGG analysis indicated that DEGs primarily participated in MAPK signaling pathway and human T-cell leukemia virus 1 infection (GSE35487, Figure 2D), osteoclast differentiation and IL-17 signaling pathway (GSE115857, Figure 3D). Simultaneously, GO analysis suggested that DEGs were primarily connected with response to nutrient levels and cellular response to extracellular/external stimulus (GSE35487, Figure 2E), regulation of hemopoiesis and leukocyte differentiation (GSE115857, Figure 3E).

### Identification of Immune-Associated Hub Genes

**FIGURE 4:**
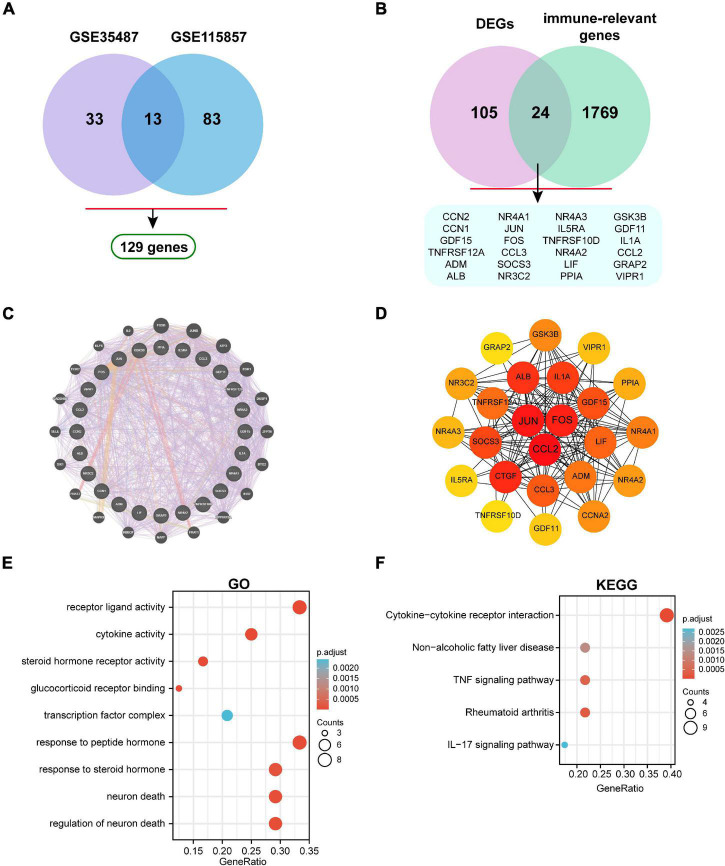
Identification of immune-associated hub genes. **(A)** Venn diagram of DEGs in GSE35487 and GSE115857. **(B)** Venn diagram of DEGs and immune-associated gene list. **(C)** The visualized gene-gene interaction network of immune-associated DEGs. **(D)** The visualized PPI network of immune-associated DEGs. **(E)** GO and **(F)** KEGG enrichment analyses of immune-associated DEGs.

A total of 129 DEGs were obtained between IgAN and healthy controls from the combination of GSE35487 and GSE115857 datasets (Figure 4A). Furthermore, the intersection was screened out between DEGs and the immune-associated gene list by means of a Venn diagram, which contained 24 genes (Figure 4B). The detailed genes information involving in DEGs, immune-relevant genes, and immune-associated hub genes can be inquired in the Supplementary Material. Subsequently, the gene-gene interaction network of immune-associated hub genes was established utilizing Genemania database (Figure 4C). The Protein-Protein Interaction (PPI) network was analyzed and visualized utilizing Cytoscape (Figure 4D). GO and KEGG enrichment analyses of 24 genes were performed respectively (Figures 4E,F). Top 10 immune-associated hub genes selected by MCC approach were displayed by Table 1.

**TABLE 1:** The top 10 immune-associated hub genes ranked by MCC method.

Rank	Gene	MCC score
1	CCL2	5.32E + 07
2	JUN	5.32E + 07
3	FOS	5.32E + 07
4	CTGF	5.31E + 07
5	ALB	5.31E + 07
6	IL1A	5.31E + 07
7	SOCS3	5.24E + 07
8	GDF15	5.12E + 07
9	CCL3	4.44E + 07
10	LIF	4.43E + 07

### Gene Expression and Diagnostic Value Analysis of the Potential Biomarkers

**FIGURE 5:**
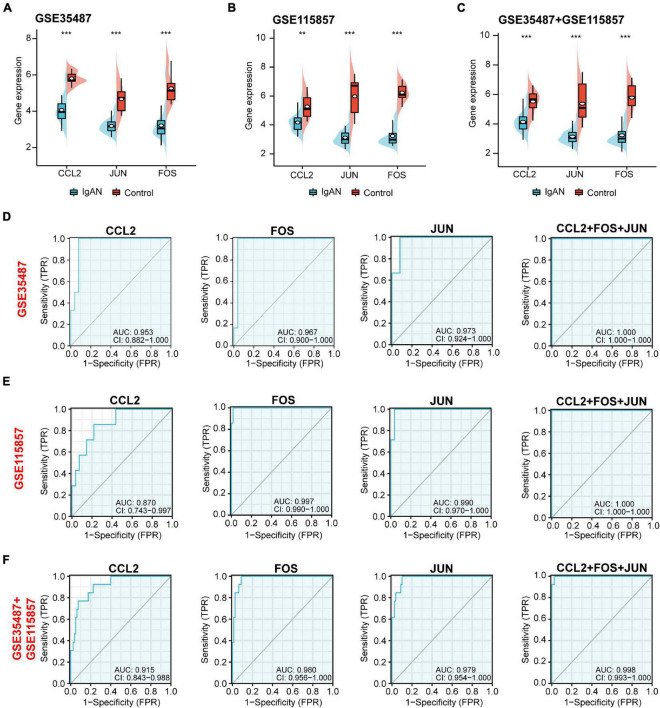
Gene expression and diagnostic value analysis of the potential biomarkers (CCL2, JUN, and FOS). The expression level of CCL2, JUN, and FOS between IgAN samples and healthy controls in **(A)** GSE35487, **(B)** GSE115857, and **(C)** the combined GSE35487-GSE115857 dataset. The diagnostic value of CCL2, FOS, JUN, and combined CCL2-FOS-JUN according to AUC value in ROC curve based on **(D)** GSE35487 (AUC = 0.953, AUC = 0.967, AUC = 0.973, and AUC = 1, respectively), **(E)** GSE115857 (AUC = 0.870, AUC = 0.997, AUC = 0.990, and AUC = 1, respectively), and **(F)** the combined GSE35487-GSE115857 dataset (AUC = 0.915, AUC = 0.980, AUC = 0.979, and AUC = 0.998, respectively). **p < 0.01 and ***p < 0.001.

Top three immune-associated hub genes (CCL2, FOS, and JUN) were defined as the potential biomarkers. We first evaluated the expression level of the three biomarkers between IgAN and healthy controls. As shown in Figures 5A–C, these genes were all remarkably downregulated in IgAN. AUC is characterized with sensitivity and specificity, which is generally utilized to indicate the intrinsic effectiveness of diagnostic tests (Kumar and Indrayan, 2011). The results suggested that CCL2, FOS, and JUN had a high diagnostic value according to AUC value in ROC curve based on GSE35487 (AUC = 0.953, AUC = 0.967, AUC = 0.973, respectively) (Figure 5D), GSE115857 (AUC = 0.870, AUC = 0.997, AUC = 0.990, respectively) (Figure 5E), and combined GSE35487-GSE115857 (AUC = 0.915, AUC = 0.980, AUC = 0.979, respectively) (Figure 5F). Noteworthy, the diagnostic value of combined the three genes CCL2-FOS-JUN was higher (AUC reached almost 1), which indicated that the three genes could serve as potential diagnostic biomarkers for IgAN. In summary, CCL2, FOS and JUN performed well in distinguishing IgAN patients and healthy individuals.

### Identification of IgAN Subgroups and Gene Expression Analysis

**FIGURE 6:**
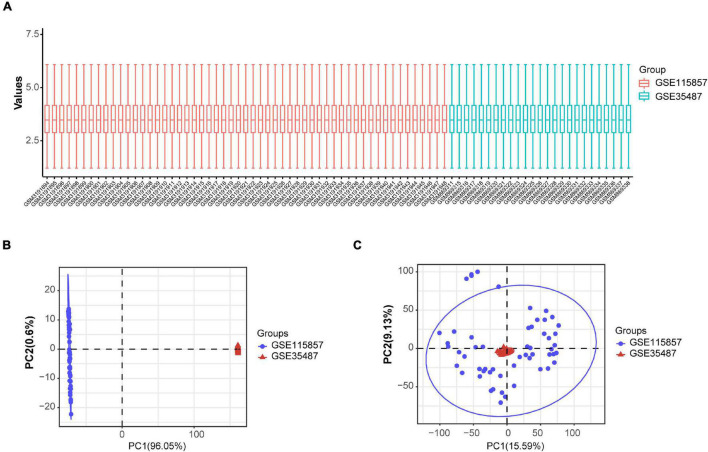
Standardization and batch effect removal for GSE35487 and GSE115857. **(A)** Box plot after data standardization. **(B)** PCA results before batch removal for GSE35487 and GSE115857. **(C)** PCA results after batch removal for GSE35487 and GSE115857.

**FIGURE 7:**
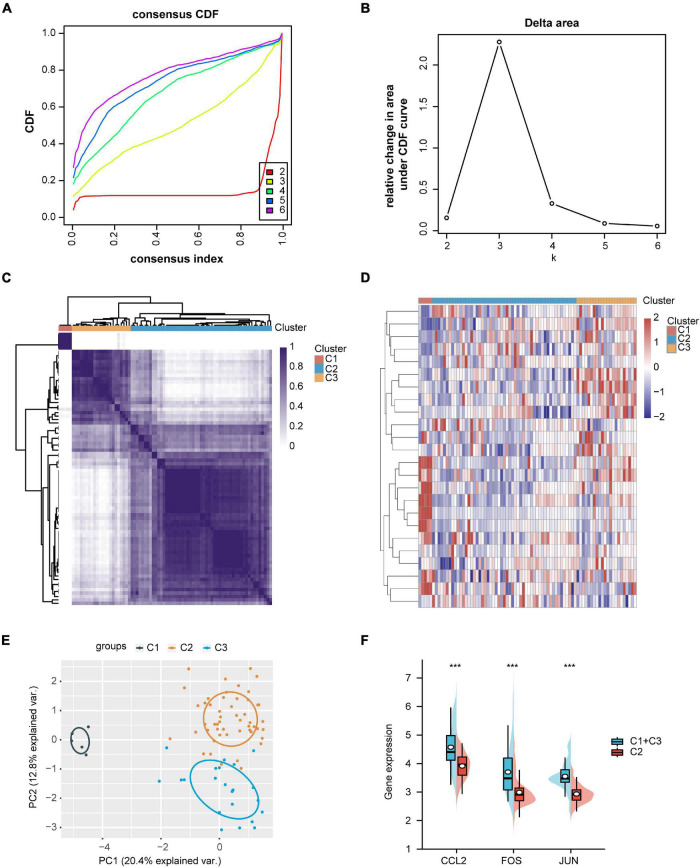
Identification of IgAN subgroups and gene expression analysis. **(A)** Consensus clustering of cumulative distribution function (CDF) for *k* = 2–6. **(B)** Elbow plot displays relative change in area under CDF curve. **(C)** Heatmap depicting consensus clustering solution (*k* = 3). **(D)** Heatmap of immune-associated DEGs expression in three subgroups, red represents high expression and blue represents low expression. **(E)** Principal component analysis (PCA) of three IgAN subgroups. **(F)** The expression levels of CCL2, FOS, and JUN in the different subgroups of IgAN. ***p < 0.001.

The expression data from GSE35487 and GSE115857 were normalized and merged, and a total of 80 IgAN samples were collected (Figure 6A). The PCA results indicated that the two datasets could be used as a batch of data for subsequent analysis through batch removal (Figures 6B,C). To identify the subgroups of IgAN, we conducted unsupervised clustering according to 24 immune-associated hub genes and *k* = 3 seemed to be an adequate selection. Then, 80 IgAN patients were ultimately clustered into three distinct subgroups, identified as cluster 1 (C1, *n* = 5), cluster 2 (C2, *n* = 53), and cluster 3 (C3, *n* = 22) (Figures 7A–D). The three clusters of IgAN had remarkably different populations in PCA (Figure 7E). We further investigated the expression of the three diagnostic biomarkers in the different subgroups of IgAN (Figure 7F).

### Correlation Analysis of CCL2, FOS, JUN Expression With the Clinicopathological Features

**FIGURE 8:**
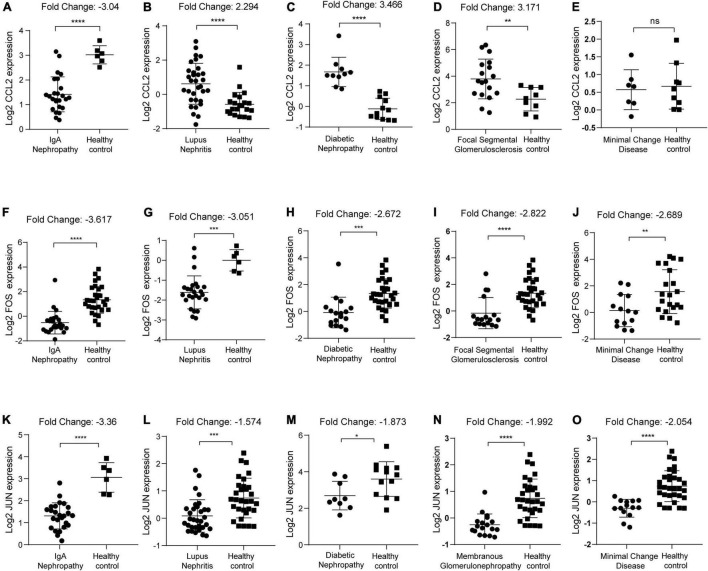
Identification of CCL2, FOS, JUN expression in kidney diseases with healthy individuals as control. **(A–E)** CCL2 expression in IgAN, lupus nephritis, diabetic nephropathy, focal segmental glomerulosclerosis, and minimal change disease, respectively. **(F–J)** FOS expression in IgAN, lupus nephritis, diabetic nephropathy, focal segmental glomerulosclerosis, and minimal change disease, respectively. **(K–O)** JUN expression in IgAN, lupus nephritis, diabetic nephropathy, membranous glomerulonephropathy, and minimal change disease, respectively. ns, no significance, *p < 0.05, **p < 0.01, ***p < 0.001, and ****p ≤ 0.0001.

We first identified the expression level of CCL2, FOS, JUN in IgAN, lupus nephritis, diabetic nephropathy, focal segmental glomerulosclerosis, minimal change disease, and membranous glomerulonephropathy, respectively. Results indicated that CCL2 was significantly downregulated in IgAN (*p* < 0.0001, Fold Change: −3.04) (Figure 8A). In contrast, CCL2 was overexpressed in lupus nephritis (*p* < 0.0001, Fold Change: 2.294), diabetic nephropathy (*p* < 0.0001, Fold Change: 3.466), focal segmental glomerulosclerosis (*p* < 0.01, Fold Change: 3.171), and minimal change disease (*p* > 0.05) (Figures 8B–E). FOS gene was downregulated in IgAN (*p* < 0.0001, Fold Change: −3.617), lupus nephritis (*p* < 0.001, Fold Change: −3.051), and diabetic nephropathy (*p* < 0.001, Fold Change: −2.672), focal segmental glomerulosclerosis (*p* < 0.0001, Fold Change: −2.822), and minimal change disease (*p* < 0.01, Fold Change: −2.689) (Figures 8F–J). Furthermore, JUN was downregulated in IgAN (*p* < 0.0001, Fold Change: −3.36), lupus nephritis (*p* < 0.001, Fold Change: −1.574), and diabetic nephropathy (*p* < 0.05, Fold Change: −1.873), membranous glomerulonephropathy (*p* < 0.0001, Fold Change: −1.992), and minimal change disease (*p* < 0.0001, Fold Change: −2.054) (Figures 8K–O). Consequently, we uncovered that there was the most significant downregulation of CCL2, FOS, JUN in IgAN according to the Fold Change.

**FIGURE 9:**
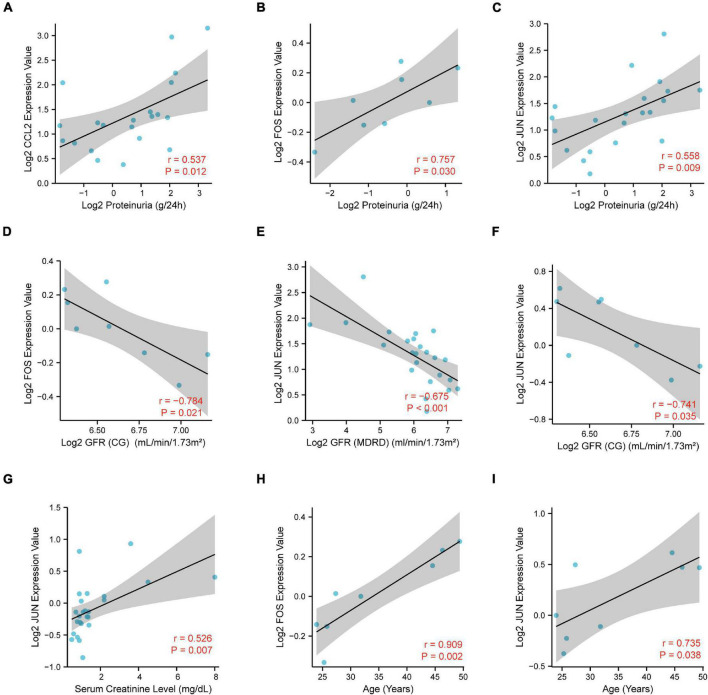
Correlation analysis of CCL2, FOS, JUN expression with the clinicopathological features, including **(A–C)** proteinuria, **(D–F)** glomerular filtration rate (GFR), **(G)** serum creatinine level, and **(H,I)** age.

To further elucidate the potential role of CCL2, FOS, JUN in IgAN, we performed the correlation analysis of CCL2, FOS, JUN expression with several clinicopathological features *via* Nephroseq database. Notably, there was a significantly positive association between CCL2/FOS/JUN expression and proteinuria (Figures 9A–C). Meanwhile, there was a significantly negative association between FOS/JUN expression and glomerular filtration rate (GFR) (Figures 9D–F). We also uncovered that JUN expression was positively linked with serum creatinine level (Figure 9G). Finally, results indicated that FOS/JUN expression positively correlated with patients’ age (Figures 9H,I). Thus, a relatively higher expression of CCL2, FOS, JUN may indicate poor renal function in the patients with IgAN and may predict the prognosis in the patients with IgAN.

## Discussion

IgAN is one of the most common types of glomerulonephritis in humans, occupying approximately 50% of primary glomerulonephritis in Asian populations (Hou et al., 2018). The feature is mainly IgA immunocomplex deposition in the mesangial region of the glomerulus with variable clinical and pathological manifestations. IgAN patients have a poor long-term prognosis, of which 25% of patients have progressed to ESRD, a more advanced state, within 20 years requiring the treatment of kidney replacement or transplantation (Magistroni et al., 2015). Since the pathogenesis of IgAN, so far, has not been fully clarified, many studies supported it as an immune-mediated disease (Rodrigues et al., 2017). Immunotherapy is one of the common treatments for immune-mediated kidney disease, but there are still many controversies about the efficacy of immunosuppressive agents in IgAN (Magistroni et al., 2015; Rodrigues et al., 2017; Holdsworth and Kitching, 2018). Renal biopsy currently serves as a gold standard for diagnosis of renal disease. Although biopsy can provide greater information, it is not suitable for repeated operation concerning high risk of invasive procedure and the case that the small biopsy specimen is unable to reflect the true status of the whole kidney (Stanley et al., 2020). Therefore, non-invasive biomarkers with high specificity and sensitivity for the early detection of IgAN are highly desired. This study aimed to explore potential molecular biomarkers for the diagnosis of IgAN.

Our findings disclosed that a total of 129 DEGs were determined between IgAN and living healthy donors from the datasets GSE115857 and GSE35487, of which 24 DEGs were associated with immune. Previous studies have shown that inflammatory reaction, glomerular and tubular fibrosis have a significant connection to the development and prognosis of IgAN (Bao et al., 2014; Guo and Liao, 2017; Zhang L. et al., 2017; Sheng et al., 2018). The MAPK pathway is also related to tubule-interstitial fibrosis in IgAN (Zhang and Li, 2004). In the present study, KEGG analysis indicated that the DEGs were mainly involved in cytokine-cytokine receptor interaction, TNF signaling pathway, and MAPK signaling pathway, which was consistent with the findings of previous studies (Anders et al., 2003; Masutani et al., 2003). These bioinformatics findings may add to the evidence that immunity participates in the process of IgAN.

CCL2, JUN, and FOS genes were eventually considered as the potential biomarkers, which had characterized the high diagnostic value of IgAN. C-C motif chemokine ligand 2 (CCL2) acts as a ligand for C-C chemokine receptor 2 (CCR2), which participates in signal transduction through binding and activating CCR2 resulting in a strong chemotactic response and mobilization of intracellular calcium ions (Paavola et al., 1998; Hemmerich et al., 1999; Jarnagin et al., 1999). Feng et al. (2018) found that exosomal CCL2 mRNA expression had an association with the severity of tubular atrophy and interstitial fibrosis, particularly the infiltration level of macrophage confirmed by renal biopsy. Moreover, urinary CCL2 exerted a vital role in predicting the prognosis for ESRD patients with IgAN (Torres et al., 2008).

FOS gene also participates in numerous biological processes, including growth, division, apoptosis, and migration (Durchdewald et al., 2009). Additionally, FOS is involving in DNA destruction, telomere injury, and neutrophil action, which correlates with the development and progression of IgAN (Jiang et al., 2016). A recent study has discovered that the FOS proteins are associated with the disappearance of podocyte foot processes (Park et al., 2014). JUN, one of the AP−1 family members, serves as a transcriptional activator participating in the regulation of proliferation, cell death, differentiation, and inflammation (Hess et al., 2004). Moreover, there has an association between the increased expression of AP-1 and IgAN (Cao et al., 2013). CCL2, JUN, and FOS exert a crucial role in IgAN, but there requires larger samples and clinical information for further exploring the potential mechanisms.

Additionally, in our study, three distinct subtypes of IgAN were identified based on the 24 immune-associated hub genes expression using unsupervised clustering. Due to the relatively small samples in C1 and C3, we combined C1 with C3 for analysis. Here, we found that CCL2, JUN, and FOS appeared a lower level of gene expression in C2 than C1 + C3. Finally, we detected a meaningful correlation between CCL2, FOS, JUN and renal function such as proteinuria, GFR, and serum creatinine level.

In light of the abovementioned approach, we identified that the combination of CCL2, JUN and FOS had high sensitivity and specificity in distinguishing IgAN patients from healthy controls. These biomarkers exhibited high AUC value, reaching even an AUC of 1.00. In summary, our study comprehensively uncovers that CCL2, JUN, and FOS may function as promising biomarkers for diagnosis of IgAN.

## Data Availability Statement

Publicly available datasets were analyzed in this study. This data can be found here: Two datasets (GSE115857 and GSE35487) from the Gene Expression Omnibus GEO database (http://www.ncbi.nlm.nih.gov/geo/).

## Author Contributions

XZ and PY conceived and designed the study. XZ wrote the manuscript. All authors contributed to data collection, analysis, visualization, figure generation, and reviewed and approved the final draft of the manuscript.

## Conflict of Interest

The authors declare that the research was conducted in the absence of any commercial or financial relationships that could be construed as a potential conflict of interest.

## Publisher’s Note

All claims expressed in this article are solely those of the authors and do not necessarily represent those of their affiliated organizations, or those of the publisher, the editors and the reviewers. Any product that may be evaluated in this article, or claim that may be made by its manufacturer, is not guaranteed or endorsed by the publisher.

## Footnotes

1
http://www.ncbi.nlm.nih.gov/geo/

2
https://www.immport.org/shared/genelists

3
http://genemania.org/

4
https://string-db.org/

## Abbreviations

AUC: area under the curve
CCL2: C-C motif chemokine ligand 2
CCR2: C-C chemokine receptor 2
DEGs: differentially expressed genes
ESRD: end-stage renal disease
Gd-IgA1: galactose-deficient IgA1
GEO: gene expression omnibus
GFR: glomerular filtration rate
GO: Gene Ontology
KEGG: Kyoto Encyclopedia of Genes and Genomes
IgAN: IgA nephropathy
MCC: maximal clique centrality
PCA: principal components analysis
PPI: protein–protein interaction
ROC: receiver operating characteristic.

## References

[B1] Amico G. (2000). Natural history of idiopathic IgA nephropathy: role of clinical and histological prognostic factors. *Am. J. Kidney Dis.* 36 227–237. 10922300 10.1053/ajkd.2000.8966

[B2] Anders H. J. Vielhauer V. Schlöndorff D. (2003). Chemokines and chemokine receptors are involved in the resolution or progression of renal disease. *Kidney Int.* 63 401–415. 10.1046/j.1523-1755.2003.00750.x 12631106

[B3] Bao H. Hu S. Zhang C. Shi S. Qin W. Zeng C. (2014). Inhibition of miRNA-21 prevents fibrogenic activation in podocytes and tubular cells in IgA nephropathy. *Biochem. Biophys. Res. Commun.* 444 455–460. 24468088 10.1016/j.bbrc.2014.01.065

[B4] Cao W. Xu J. Zhou Z. M. Wang G. B. Hou F. F. Nie J. (2013). Advanced oxidation protein products activate intrarenal renin-angiotensin system via a CD36-mediated, redox-dependent pathway. *Antioxid. Redox Signal.* 18 19–35. 22662869 10.1089/ars.2012.4603 PMC3503474

[B5] Coppo R. (2017). Biomarkers and targeted new therapies for IgA nephropathy. *Pediatr. Nephrol.* 32 725–731. 10.1007/s00467-016-3390-9 27324471

[B6] Durchdewald M. Angel P. Hess J. (2009). The transcription factor Fos: a Janus-type regulator in health and disease. *Histol. Histopathol.* 24 1451–1461. 10.14670/HH-24.1451 19760594

[B7] Feng Y. Lv L. L. Wu W. J. Li Z. L. Chen J. Ni H. F. (2018). Urinary exosomes and exosomal CCL2 mRNA as biomarkers of active histologic injury in IgA nephropathy. *Am. J. Pathol.* 188 2542–2552. 30142333 10.1016/j.ajpath.2018.07.017

[B8] Gorgi Y. Hbibi I. Sfar I. Gargueh T. Cherif M. Goucha Louzir R. (2010). Role of genetic polymorphisms in factor H and MBL genes in Tunisian patients with immunoglobulin A nephropathy. *Int. J. Nephrol. Renovasc. Dis.* 3 27–32. 21694925 10.2147/ijnrd.s8442 PMC3108773

[B9] Guo Y. Liao Y. (2017). miR-200bc/429 cluster alleviates inflammation in IgA nephropathy by targeting TWEAK/Fn14. *Int. Immunopharmacol.* 52 150–155. 10.1016/j.intimp.2017.09.002 28910745

[B10] Hemmerich S. Paavola C. Bloom A. Bhakta S. Freedman R. Grunberger D. (1999). Identification of residues in the monocyte chemotactic protein-1 that contact the MCP-1 receptor, CCR2. *Biochemistry* 38 13013–13025. 10.1021/bi991029m 10529171

[B11] Hess J. Angel P. Schorpp-Kistner M. (2004). AP-1 subunits: quarrel and harmony among siblings. *J. Cell Sci.* 117(Pt 25), 5965–5973. 15564374 10.1242/jcs.01589

[B12] Holdsworth S. R. Kitching A. R. (2018). Immune-mediated kidney disease in 2017: progress in mechanisms and therapy for immunological kidney disease. *Nat. Rev. Nephrol.* 14 76–78. 10.1038/nrneph.2017.171 29292371

[B13] Hou J. H. Zhu H. X. Zhou M. L. Le W. B. Zeng C. H. Liang S. S. (2018). Changes in the spectrum of kidney diseases: an analysis of 40,759 biopsy-proven cases from 2003 to 2014 in China. *Kidney Dis.* 4 10–19. 10.1159/000484717 29594138 PMC5848489

[B14] Jarnagin K. Grunberger D. Mulkins M. Wong B. Hemmerich S. Paavola C. (1999). Identification of surface residues of the monocyte chemotactic protein 1 that affect signaling through the receptor CCR2. *Biochemistry* 38 16167–16177. 10.1021/bi9912239 10587439

[B15] Jiang H. Liang L. Qin J. Lu Y. Li B. Wang Y. (2016). Functional networks of aging markers in the glomeruli of IgA nephropathy: a new therapeutic opportunity. *Oncotarget* 7 33616–33626. 27127888 10.18632/oncotarget.9033 PMC5085107

[B16] Kidney Disease: Improving Global Outcomes [KDIGO] Glomerular Diseases Work Group (2021). KDIGO 2021 clinical practice guideline for the management of glomerular diseases. *Kidney Int.* 100 S1–S276. 10.1016/j.kint.2021.05.021 34556256

[B17] Kumar R. Indrayan A. (2011). Receiver operating characteristic (ROC) curve for medical researchers. *Indian Pediatr.* 48 277–287. 10.1007/s13312-011-0055-4 21532099

[B18] Lee H. Hwang J. H. Paik J. H. Ryu H. Kim D. Chin H. (2014). Long-term prognosis of clinically early IgA nephropathy is not always favorable. *BMC Nephrol.* 15:94. 10.1186/1471-2369-15-94 24946688 PMC4070337

[B19] Leung J. C. Poon P. Y. Lai K. N. (1999). Increased sialylation of polymeric immunoglobulin A1: mechanism of selective glomerular deposition in immunoglobulin A nephropathy. *J. Lab. Clin. Med.* 133 152–160. 10.1016/s0022-2143(99)90008-2 9989767

[B20] Leung J. C. Tang S. C. Chan L. Y. Chan W. L. Lai K. N. (2008). Synthesis of TNF-alpha by mesangial cells cultured with polymeric anionic IgA–role of MAPK and NF-kappaB. *Nephrol. Dial. Transplant.* 23 72–81. 10.1093/ndt/gfm581 17898021

[B21] Magistroni R. D. Agati V. D. Appel G. B. Kiryluk K. (2015). New developments in the genetics, pathogenesis, and therapy of IgA nephropathy. *Kidney Int.* 88 974–989. 10.1038/ki.2015.252 26376134 PMC4653078

[B22] Masutani K. Miyake K. Nakashima H. Hirano T. Kubo M. Hirakawa M. (2003). Impact of interferon-gamma and interleukin-4 gene polymorphisms on development and progression of IgA nephropathy in Japanese patients. *Am. J. Kidney Dis.* 41 371–379. 10.1053/ajkd.2003.50046 12552499

[B23] Paavola C. D. Hemmerich S. Grunberger D. Polsky I. Bloom A. Freedman R. (1998). Monomeric monocyte chemoattractant protein-1 (MCP-1) binds and activates the MCP-1 receptor CCR2B. *J. Biol. Chem.* 273 33157–33165. 9837883 10.1074/jbc.273.50.33157

[B24] Park H. J. Kim J. W. Cho B. S. Chung J. H. (2014). Association of FOS-like antigen 1 promoter polymorphism with podocyte foot process effacement in immunoglobulin A nephropathy patients. *J. Clin. Lab. Anal.* 28 391–397. 10.1002/jcla.21699 24652774 PMC6807477

[B25] Parker H. S. Leek J. T. Favorov A. V. Considine M. Xia X. Chavan S. (2014). Preserving biological heterogeneity with a permuted surrogate variable analysis for genomics batch correction. *Bioinformatics* 30 2757–2763. 10.1093/bioinformatics/btu375 24907368 PMC4173013

[B26] Ritchie M. E. Phipson B. Wu D. Hu Y. Law C. W. Shi W. (2015). limma powers differential expression analyses for RNA-sequencing and microarray studies. *Nucleic Acids Res.* 43:e47. 10.1093/nar/gkv007 25605792 PMC4402510

[B27] Rodrigues J. C. Haas M. Reich H. N. (2017). IgA nephropathy. *Clin. J. Am. Soc. Nephrol.* 12 677–686. 28159829 10.2215/CJN.07420716 PMC5383386

[B28] Schena F. P. Nistor I. (2018). Epidemiology of IgA nephropathy: a global perspective. *Semin. Nephrol.* 38 435–442. 30177015 10.1016/j.semnephrol.2018.05.013

[B29] Sheng X. Zuo X. Liu X. Zhou Y. Sun X. (2018). Crosstalk between TLR4 and Notch1 signaling in the IgA nephropathy during inflammatory response. *Int. Urol. Nephrol.* 50 779–785. 10.1007/s11255-017-1760-2 29230705

[B30] Stanley S. Vanarsa K. Soliman S. Habazi D. Pedroza C. Gidley G. (2020). Comprehensive aptamer-based screening identifies a spectrum of urinary biomarkers of lupus nephritis across ethnicities. *Nat. Commun.* 11:2197. 32366845 10.1038/s41467-020-15986-3 PMC7198599

[B31] Su X. Lv J. Liu Y. Wang J. Ma X. Shi S. (2017). Pregnancy and kidney outcomes in patients with IgA nephropathy: a cohort Study. *Am. J. Kidney Dis.* 70 262–269. 10.1053/j.ajkd.2017.01.043 28320554

[B32] Suzuki H. (2019). Biomarkers for IgA nephropathy on the basis of multi-hit pathogenesis. *Clin. Exp. Nephrol.* 23 26–31. 10.1007/s10157-018-1582-2 29740706 PMC6344607

[B33] Torres D. D. Rossini M. Manno C. Mattace-Raso F. D’Altri C. Ranieri E. (2008). The ratio of epidermal growth factor to monocyte chemotactic peptide-1 in the urine predicts renal prognosis in IgA nephropathy. *Kidney Int.* 73 327–333. 10.1038/sj.ki.5002621 17943082

[B34] Wilkerson M. D. Hayes D. N. (2010). ConsensusClusterPlus: a class discovery tool with confidence assessments and item tracking. *Bioinformatics* 26 1572–1573. 10.1093/bioinformatics/btq170 20427518 PMC2881355

[B35] Yang B. Zhang J. Liu X. Huang Z. Su Z. Liao Y. (2018). Genetic polymorphisms in HLA-DP and STAT4 are associated with IgA nephropathy in a Southwest Chinese population. *Oncotarget* 9 7066–7074. 10.18632/oncotarget.23829 29467950 PMC5805536

[B36] Zhang D. Xie M. Yang X. Zhang Y. Su Y. Wang Y. (2017). Determination of IL-1B (rs16944) and IL-6 (rs1800796) genetic polymorphisms in IgA nephropathy in a northwest Chinese Han population. *Oncotarget* 8 71750–71758. 10.18632/oncotarget.17603 29069743 PMC5641086

[B37] Zhang L. Han C. Ye F. He Y. Jin Y. Wang T. (2017). Plasma gelsolin induced glomerular fibrosis via the TGF-β1/Smads signal transduction pathway in IgA nephropathy. *Int. J. Mol. Sci.* 18:390. 10.3390/ijms18020390 28208683 PMC5343925

[B38] Zhang M. Li X. M. (2004). [Relationship of mitogen-activated protein kinases activation with transdifferentiation of renal tubular epithelial cells in patients with IgA nephropathy]. *Zhonghua Yi Xue Za Zhi* 84 898–903. 15329273

